# Far Infrared Irradiation Enhances Nutraceutical Compounds and Antioxidant Properties in *Angelica gigas* Nakai Powder

**DOI:** 10.3390/antiox7120189

**Published:** 2018-12-11

**Authors:** Md Obyedul Kalam Azad, Jing Pei Piao, Cheol Ho Park, Dong Ha Cho

**Affiliations:** 1 College of Biomedical Science, Kangwon National University, Chuncheon 24341, Korea; azadokalam@gmail.com (M.O.K.A.); jinchwu@hotmail.com (J.P.P.); chpark@kangwon.ac.kr (C.H.P.); 2 Head of Research and Technology, Rentia Plant Factory, Chuncheon 24341, Korea

**Keywords:** AGN, FIR, phenolic, flavonoid, antioxidant capacity

## Abstract

The aim of this study was to investigate the effect of far infrared irradiation (FIR) on nutraceutical compounds, viz. total phenolic content, total flavonoids, and antioxidant capacity, of *Angelica gigas* Nakai (AGN). The FIR treatment was applied for 30 min with varied temperatures of 120, 140, 160, 180, 200, 220, and 240 °C. Results showed that FIR increased total phenolic and flavonoid content in AGN at 220 °C. The HPLC results revealed higher quantities of decursin (62.48 mg/g) and decursinol angelate (41.51 mg/g) at 220 °C compared to control (38.70 mg/g, 27.54 mg/g, respectively). The antioxidant capacity of AGN was also increased at 220 °C, as measured by 1,1-diphenyl-2-picrylhydrazyl (DPPH), ferric reducing antioxidant power (FRAP), and the phosphomolybdenum (PPMD) method. A further increase of the FIR temperature caused a reduction of compound content. In addition, the results also showed a strong correlation between phenolic content and antioxidant properties of AGN powder. These findings will help to further improve the nutraceutical profile of AGN powder by optimizing the FIR conditions.

## 1. Introduction

*Angelica gigas* Nakai (AGN) is one of the most important traditional herbal medicinal plants in Korea. AGN possesses abundant nutraceutical properties, with strong antioxidant capacity [1]. The roots of AGN contain coumarin derivatives decursin (D) and decursinol angelate (DA) [2] which have several pharmacological properties such as anti-amnestic activity [3] anti-bacterial action [4], anti-allergic effect [5], and anti-tumor activity [6]. AGN is mainly used for the treatment of menopausal syndromes in Korea; therefore, it is called ‘female ginseng’ [7]. 

Plant bioactive compounds have a strong intermolecular covalent bond with large molecular weight. This complex molecular structure makes them less functional [8]. Many methods, including mechanical, chemical, and radiation approaches, are being employed to improve the plant antioxidant profiles through the stretching and bending of molecules. Among those, far infrared irradiation (FIR) is an efficient and convenient method to liberate the molecules from their complex crystalline structure and reduce intermolecular energy [9]. FIR is a subdivision of the electromagnetic spectrum which is used in the food processing industry to induced biological activities and prevent food quality degradation [10]. 

FIR has the capacity to transfer heat through molecular vibration to the center of the materials without degrading the constituent of the molecules. FIR cleaves covalent bonds and liberates antioxidants such as phenolic acids, flavonoids, tannins, and carotenoids [7,10].

It is reported that FIR increases the quantities of nutraceutical compounds, while improving antioxidant property, anti-inflammatory, and inhibitory activity in the A549 Cell line in *Chrysanthemum indicum* L. [11]. In particular, FIR enhances phenolic content and antioxidant capacity of buckwheat flour [12], sprouting bean flour [13], citrus cack [14], and rice hull [15]. However, to the best of our knowledge, there are no reports published on the effect of FIR on nutraceutical compounds in AGN. Therefore, the objective of this study was to induce nutraceutical compounds and increase antioxidant capacity through the application of FIR treatment in AGN powder. 

## 2. Materials and Methods

### 2.1. Application of FIR Treatment and Preparation of AGN Extract

*Angelica gigas* Nakai (AGN) was purchased from Pyeongchang local market, Korea. AGN was dried in an oven at 50 °C for 24 h and powdered using a grinder. The powder was meshed using a 200 µm sieve to obtain a uniform particle size of the powder. Two grams of powder were mixed with 4 mL of water in a glass petri dish and exposed to an FIR dryer (HKD-10; Korea Energy Technology, Seoul, Korea) at 120, 140, 160, 180, 200, 220 and 240 °C for 30 min. Afterward, 1 g of the treated powder and control sample (without FIR application) was suspended in 100 mL of 80% ethanol and kept over-night in a shaker at room temperature. The extracts were filtered through Advantech 5B filter paper (Tokyo Roshi Kaisha Ltd., Saitama, Japan) and dried using a vacuum rotatory evaporator (EYLA N-1000, Tokyo, Japan) in 40 °C water bath to obtain crude extracts. The crude extracts were freeze-dried to achieve a moisture content of <3–5%. Dried crude extracts were diluted using 80% ethanol to prepare 1000 mg/L stock solution and kept at −20 °C for further analysis. 

### 2.2. Estimation of Total Phenolic Content

Total phenolic (TP) content of FIR treated and control AGN samples were determined by the Folin Ciocalteu assay [16]. In brief, a sample aliquot of 1 mL of extract (1 mg/mL) was added to a test tube containing 0.2 mL of phenol reagent (1 N). The volume was increased by adding 1.8 mL of deionized water and the solution was vortexed and left for 3 min for reaction. Furthermore, 0.4 mL of Na_2_CO_3_ (10% in water, v/v) was added and the final volume (4 mL) was adjusted by adding 0.6 mL of deionized water. The absorbance was measured at 725 nm after incubation for 1 h at room temperature. The TP content was calculated from a calibration curve using gallic acid and expressed as mg of gallic acid equivalent (GAE) per g dry weight (dw).

### 2.3. Determination of Total Flavonoid Content

The total flavonoid content (TF) content was quantified according to Ghimeray et al. [12] with slight modifications. Shortly, a 0.5 mL aliquot of the sample (1 mg/mL) was mixed with 0.1 mL of 10% aluminum nitrate and 0.1 mL of potassium acetate (1 M) solution. To this mixture, 3.3 mL of distilled water was added to make the total volume of 4 mL. The mixture was vortexed and incubated for 40 min. The TF content was measured using a spectrophotometer (UV-1800 240 V, Shimadzu Corporation, Kyoto, Japan) at 415 nm. The TF content was expressed as mg/g coumarin equivalents on a dry weight basis.

### 2.4. HPLC Analysis of AGN Extract with or without FIR Treatments 

An HPLC system (CBM-20A, Shimadzu Co, Ltd., Kyoto, Japan) with two gradient pump systems (LC-20AT, Shimadzu), a C18 column (Kinetex, 100 × 4.6 mm, 2.6 micron, Phenomenex), an auto-sample injector (SIL-20A, Shimadzu), a UV detector (SPD-10A, Shimadzu) and a column oven (35 °C, CTO-20A, Shimadzu) were used to analyze D and DA. Solvent A was water with 0.4% formic acid, and solvent B was acetonitrile. A gradient elution was used (0–15 min, 33–45% B; 15–30 min, 45–55% B; 30–40 min, 55–80% B; 40–45 min, 80–33% B). The flow rate was 1.0 mL/min, the injection volume was 10 μL, and the detection wavelength was 329 nm. The standard samples for the D and DA analysis were prepared at concentrations of 10, 20, 40, 60 and 80 μg/mL. As D and DA are the main active ingredients of the AGN therefore, they were separated and then quantified by HPLC.

### 2.5. Antioxidant Capacity Analysis

#### 2.5.1. DPPH Free Radical Scavenging Capacity 

The antioxidant capacity was determined on the basis of the scavenging activity of the stable 2, 2-diphenyl-1 picryl hydrazyl (DPPH) free radical according to methods described by Braca et al. [17]. The DPPH solution was prepared (5.914 mg of DPPH powder dissolved in 100 mL of methanol) to maintain an absorbance range of 1.1–1.3 by spectrophotometer. Briefly, 1 mL of stock solution was added to 3 mL of DPPH solution. The blank sample was prepared with 1 mL of distilled water instead of stock extract in 3 mL of DPPH solution. The mixture was shaken vigorously and left to stand at room temperature in the dark for 30 min. The absorbance was measured at 517 nm using a spectrophotometer (UV-1800 240 V, Shimadzu Corporation, Kyoto, Japan). The percent inhibition activities of the treated and control AGN samples were calculated against a blank sample using the following equation: 

Inhibition (%) = [(blank sample − extract sample)/blank sample] × 100.

#### 2.5.2. Ferric Reducing Antioxidant Power Assay (FRAP) 

The reducing power of the samples was estimated according to the FRAP assay as described by Yu et al. [18]. In brief, 1 mL of stock solution (1 mg/mL) was mixed with 1 mL of 0.2 M phosphate buffer maintaining a pH of 6.6. The mixture was then incubated at 50 °C for 20 min. After incubation, 1 mL of trichloro-acetic acid (TCA) was added to the solution and centrifuged at 3000 rpm for 10 min. The collected supernatant was diluted with distilled water at 1:1 ratio. Finally, 0.25 mL of 0.1% ferric chloride was added and the absorbance was measured at 700 nm by a spectrophotometer.

#### 2.5.3. Phosphomolybdenum Method (PPMD)

The total antioxidant capacity of AGN stock solution (1 mg/mL) was assayed according to the PPMD method described by Prieto et al. [19]. In brief, 1 mL of stock solution was added with 3 mL of 0.6 M sulfuric acid, 28 mM sodium phosphate and 4 mM ammonium molybdate solution. The reaction mixture was incubated at 95 °C for 150 min. The absorbance of the mixture was measured at 695 nm by spectrophotometer against a blank. The antioxidant capacity was expressed as the absorbance of the sample. 

## 3. Statistical Analysis

All data were expressed as mean ± SD of triplicate measurements. The obtained results were compared among the different FIR temperature using a paired *t*-test in order to observe the significant differences at the level of 5%. The paired *t*-test between the mean values of the treated samples and control were analyzed by MINITAB (version 16.0).

## 4. Results and Discussion 

### 4.1. Effect of FIR Irradiation on Total Phenolic and Flavonoid Contents of AGN Powder 

**Figure 1:**
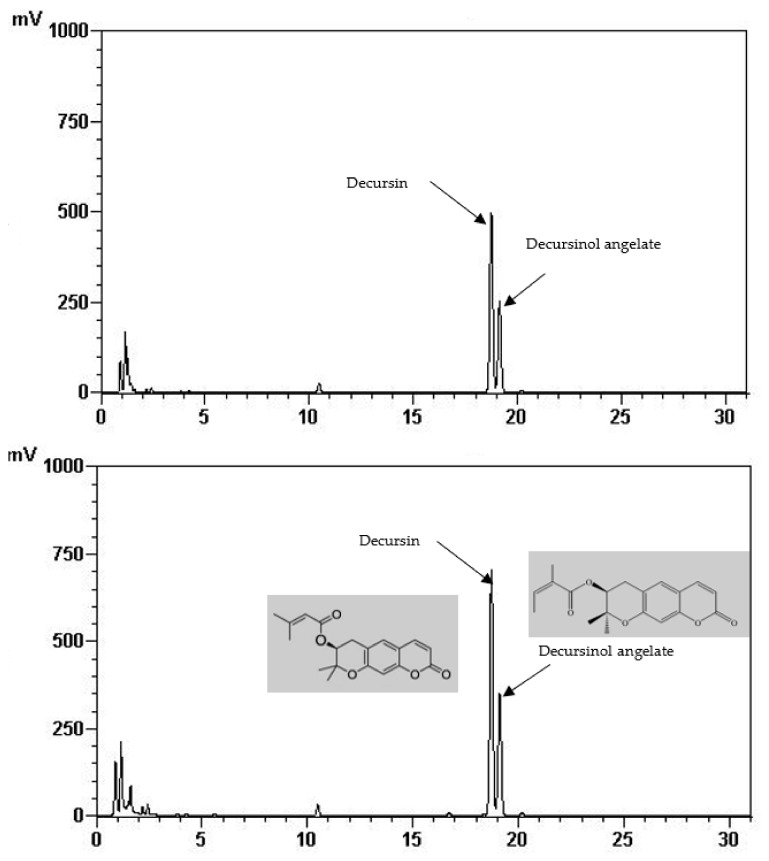
HPLC chromatogram of decursin and decursinol angelate of control (above) and treated AGN at 220 °C (down).

Total phenolic and total flavonoid content of the FIR treated and control AGN was demonstrated in Table 1. It is shown that total phenolic (22.65 mg/g) and total flavonoid content (7.87 mg/g) was significantly increased at 220 °C of FIR treatment which is two times higher than of the control (12.75 mg/g, 2.51 mg/g, respectively). In the same way, it is shown in Table 2 and Figure 1 that the quantities of the main active ingredients decursin (62.48 mg/g) and decursinol angelate (41.51 mg/g) were about two and 1.5 times higher at 220 °C than the control (38.70 mg/g, 27.94 mg/g, respectively). 

It is stated that FIR is magnificently applied in the drying of various food materials [20]. FIR creates internal heating via molecular vibration of the materials i.e., molecules absorb the radiation of certain wavelengths and energy, causing excited vibration [21]. FIR significantly induced plant secondary plant metabolites through the breakdown of a covalent bond of long-chain polymers [22]. Previous studies observed that FIR application increased phenolic content in soybean sprout powder [13], rice hull [15], ginseng, garlic, tomato, grapes, and onion [22]. In our study, the highest phenolic and flavonoid compounds, including D and DA, were attained at 220 °C of FIR treatment. Since D and DA have several pharmacological properties, therefore, FIR would be a suitable stimuli to enhance these compounds. 

Previously, Ghimeray et al. [12] obtained the highest quantities of total phenols, total flavonoids, and quercetin content of buckwheat at 120 °C for 60 min of the FIR treatment. On the other hand, Azad et al. [13] observed the highest phenolic and isoflavonoid content in soybean sprout powder at 120 °C with an exposure time of 120 min. In our study, the bioactive compounds content decreased as the FIR temperature was increased. The FIR treatment at 220 °C found to be more adventurous in this study. However, Adak et al. [23] showed that the content of phenolic compounds and anthocyanins in strawberries was reduced when the FIR temperature was above 80 °C. Therefore, it might be assumed that the optimum temperature and exposure time of the FIR depends on the plant materials.

### 4.2. Effect of FIR Irradiation on Antioxidant Capacity of AGN Powder 

Plant bioactive compounds have an enormous antioxidant capacity due to their redox properties which allow them to act as reducing agents, hydrogen donators, metal chelators and single oxygen quenchers [24].

The effect of FIR on the free radical antioxidant capacity of the AGN powder was measured by the DPPH, FRAP and PPMD method. DPPH is a stable free radical compound widely used to test the free radical scavenging ability of various materials [25]. FRAP measures the reducing potential of an antioxidant reacting with a ferric tripyridyltriazine complex, producing a colored ferrous tripyridyltriazine. PPMD is based on the reduction of Mo (VI) to Mo (V) by the sample analyte and the subsequent formation of a green phosphate/Mo(V) complex [19]. 

**Figure 2:**
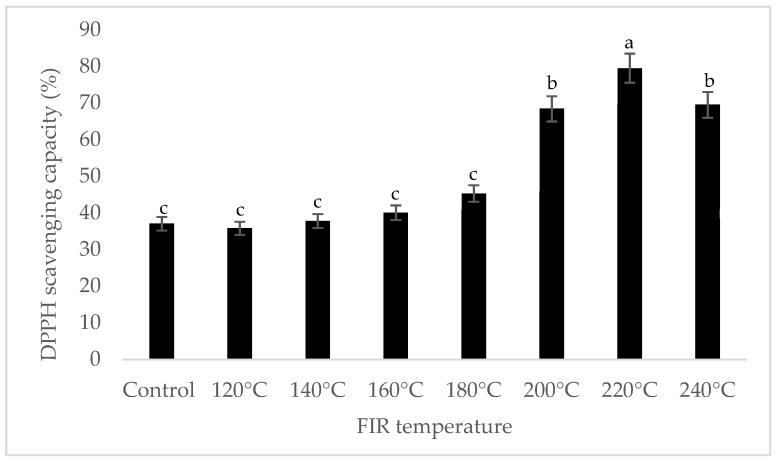
DPPH free radical scavenging activity of *A. gigas* Nakai treated by FIR irradiation. Each value is expressed as the mean ± SD (*n* = 3). Different lowercase letters within the row indicate significant differences (*p* <0.05) according to ANOVA. FIR: far infrared irradiation.

**Figure 3:**
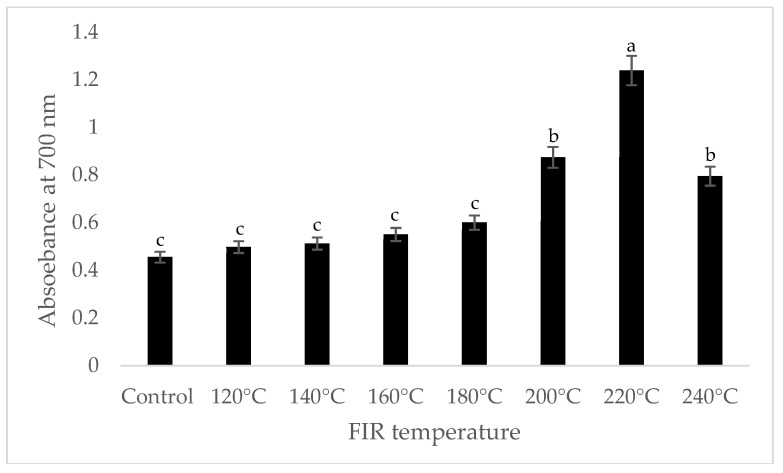
Reducing power of *A. gigas* Nakai treated by FIR irradiation (FRAP assay). Values are the mean ± SD (*n* = 3). Different lowercase letters within the row indicate significant differences (*p* <0.05) according to ANOVA. FIR: far infrared irradiation.

**Figure 4:**
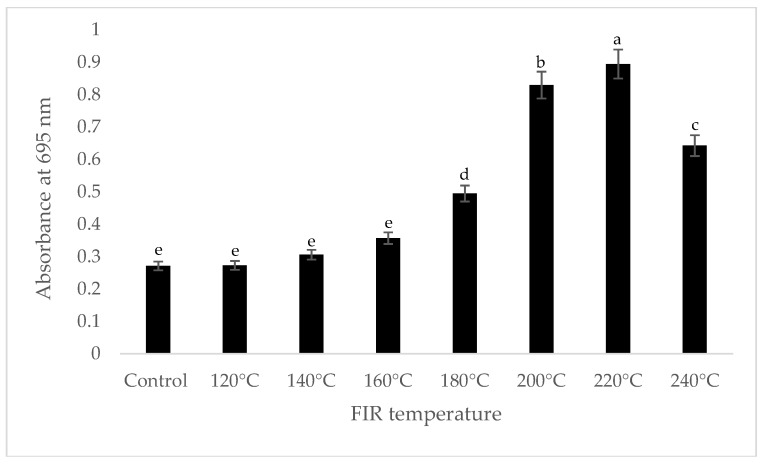
Total antioxidant capacity of *A. gigas* Nakai treated by FIR irradiation (PPMD assay). Values are the mean ± SD (*n* = 3). Different lowercase letters within the row indicate significant differences (*p* <0.05) according to ANOVA. FIR: far infrared irradiation.

It was shown that the antioxidant capacity of the AGN was increased by FIR treatment. Antioxidant capacity was found to be two times higher at 220 °C (79%) of FIR compared to control (37%), according to the DPPH analysis. In the FRAP method, antioxidants cause the reduction of the Fe3+/ferricyanide complex to the ferrous form and activity is measured as the increase in the absorbance at 700 nm. In this assay, the yellow color of the test solution changes to various shades of green and blue depending on the reducing power of antioxidant samples [26]. In FRAP assay, the absorbance was three times higher at 220 °C of FIR treatment compared to control. On the other hand, the absorbance was four times higher in treated AGN at 220 °C compared to control measured by PPMD. The reducing power of the treated sample increased with increasing concentrations in a strongly linear manner. It is clearly shown that antioxidant properties of the AGN powder were increased at 220 °C FIR in all analytical methods i.e., DPPH, FRAP, and PPMD (Figure 2, Figure 3 and Figure 4).

Previous studies showed that FIR increased the antioxidant capacity of plant food materials [12,13,15]. It was also reported that total phenolic content and antioxidant capacity has a highly significant linear correlation [23]. The increases in antioxidant activity of treated samples is due to the increase of the total polyphenol and flavonoid compounds [27].

**Figure 5:**
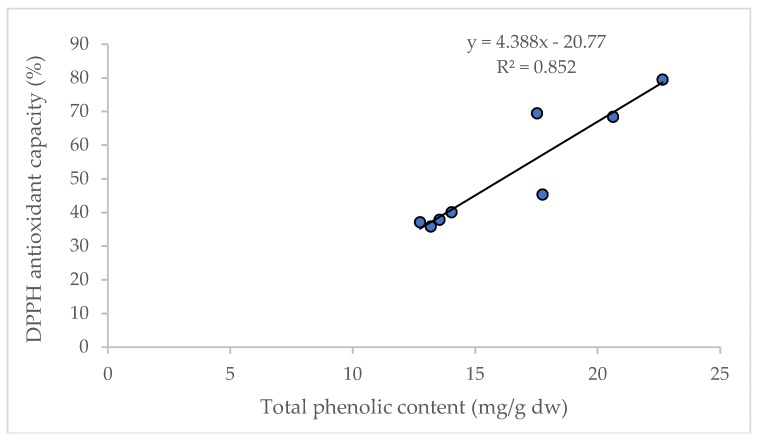
Linear regression between total phenolic content and antioxidant capacity (DPPH assay).

It is reported that D and DA are not directly correlated to the total antioxidant capacity in AGN; however, the increased content of D and DA improve the pharmacological profile [27]. Significant correlations between the content of total phenolic compounds and antioxidant capacity (R^2^ = 0.852) were observed in this study (Figure 5), and are supported by the findings of Cai et al. [28] and Djeridane et al. [29]. 

## 5. Conclusions 

Our results have demonstrated that FIR should be considered as suitable stimuli to enhance nutraceutical compounds and preserved antioxidant properties in AGN powder. The optimum FIR condition obtained for AGN is at 220 °C for 30 min. A significant linear correlation was found between phenolic content and antioxidant properties of AGN. In the current study, only FIR temperature is optimized; however, duration of FIR treatment needs to be optimized in order to attain the highest content of nutraceutical properties from AGN powder. 

## Figures and Tables

**Table 1:** Total phenolic (TP) and total flavonoid (TF) content of *Angelica gigas* Nakai treated by FIR irradiation.

FIR Treatment	TP (mg/g GAE dw)	TF (mg/g CUE dw)
Control	12.75 ± 0.25 e	2.51 ± 0.07 c
120 °C	13.19 ± 0.26 d	2.60 ± 0.05 c
140 °C	13.54 ± 0.54 d	2.73 ± 0.11 c
160 °C	14.03 ± 0.28 d	2.76 ± 0.08 c
180 °C	17.75 ± 0.53 c	3.94± 0.07 c
200 °C	20.63 ± 0.41 b	5.21 ± 0.15 b
220 °C	22.65 ± 0.22 a	7.87 ± 0.14 a
240 °C	17.53 ± 0.35 c	5.55 ± 0.16 b

Each value is expressed as the mean ± SD (*n* = 3). Values labeled with different letters in a column are significantly different (*p* < 0.05). GAE: gallic acid equivalent; CUE: coumarin equivalent; dw: dry weight.

**Table 2:** HPLC quantification of decursin and decursinol angelate content of *A. gigas* Nakai treated by FIR irradiation.

FIR Treatment	Decursin (mg/g dw)	Decursinol Angelate (mg/g dw)
Control	38.70 ± 2.11 d	27.94 ± 1.28 c
120 °C	52.89 ± 1.89 c	32.05 ± 0.92 bc
140 °C	53.12 ± 1.55 b	32.14 ± 1.31 bc
160 °C	53.52 ± 1.05 b	32.28 ± 0.95 bc
180 °C	54.27 ± 1.59 b	33.51 ± 1.07 b
200 °C	55.01 ± 1.62 b	34.21 ± 0.99 b
220 °C	62.48 ± 2.38 a	41.51 ± 1.42 a
240 °C	56.32 ± 1.43 b	36.05 ± 1.32 b

Each value is expressed as the mean ± SD (*n* = 3). Values labeled with different letters in a column are significantly different (*p* < 0.05).
